# The unexhausted potential of *E. coli*

**DOI:** 10.7554/eLife.05826

**Published:** 2015-03-25

**Authors:** Zachary D Blount

**Affiliations:** Department of Microbiology and Molecular Genetics, Michigan State University, East Lansing, United States; BEACON Center for the Study of Evolution in Action, East Lansing, United States

**Keywords:** the natural history of model organism, model organism, ecology, natural history, microbiome, pathogen, *E. coli*

## Abstract

*E. coli*'s hardiness, versatility, broad palate and ease of handling have made it the most intensively studied and best understood organism on the planet. However, research on *E.coli* has primarily examined it as a model organism, one that is abstracted from any natural history. But *E. coli* is far more than just a microbial lab rat. Rather, it is a highly diverse organism with a complex, multi-faceted niche in the wild. Recent studies of ‘wild’ *E. coli* have, for example, revealed a great deal about its presence in the environment, its diversity and genomic evolution, as well as its role in the human microbiome and disease. These findings have shed light on aspects of its biology and ecology that pose far-reaching questions and illustrate how an appreciation of *E. coli*'s natural history can expand its value as a model organism.

**DOI:** http://dx.doi.org/10.7554/eLife.05826.001

## Introduction

In 1884, the German microbiologist and pediatrician Theodor Escherich began a study of infant gut microbes and their role in digestion and disease. During this study, he discovered a fast-growing bacterium that he called *Bacterium coli commune*, but which is now known as the biological rock star that is *Escherichia coli* (Escherich, 1988; Shulman et al., 2007; Zimmer, 2008). *E. coli*'s meteoric rise and exalted status in biology stem from how easy it is to find and work with. Hardy, non-pathogenic, and versatile strains that grow quickly on many different nutrients can be isolated from virtually any human. These traits made *E. coli* a mainstay in microbiology teaching lab collections. Consequently, when early 20^th^ century microbiologists cast about for a model organism, *E. coli* was one of the most widely available choices.

Those who chose to work with *E. coli* included Bordet and Ciuca (1921), Werkman (1927), Wollman (1925), Wollman and Wollman (1937) and Bronfenbrenner and Korb (1925), Bronfenbrenner (1932), who between them performed groundbreaking studies on bacterial physiology, viruses, and genetics (Daegelen et al., 2009). By the 1940s, its use in many foundational studies firmly established *E. coli* as *the* bacterial model organism of choice, making it the obvious organism to work with at the onset of the molecular biology revolution in the 1950s. As a result*,* it became the organism in which the most basic aspects of life, including the genetic code, transcription, translation, and replication, were first worked out (Crick et al., 1961; Nirenberg et al., 1965; see Judson, 1996 for an excellent history of early molecular biology and *E. coli*'s role in it). The resulting knowledge and molecular methods for investigating and manipulating its biology have since led to *E. coli*'s prominence in academic and commercial genetic engineering, pharmaceutical production, and experimental microbial evolution (see Box 1 for a glossary of specialist terms used in this article ), not to mention the biotechnology industry, which contributed $500 billion to the global economy in 2011 (Cohen et al., 1973, Schaechter and Neidhardt, 1987; Lenski, 2004; Bruschi et al., 2011; Kamionka, 2011; Huang et al., 2012; Kawecki et al., 2013). It is not hyperbole to say that *E. coli* is now the most important model organism in biology (Zimmer, 2008; see Box 2).

10.7554/eLife.05826.002

Box 1. **Glossary**

**Accessory genes**—Genes that are not among the invariant core genome of a microbe, and are thus not present in all strains of a given species. Accessory genes are thought to improve an organism's fitness in a particular environmental or ecological context.

**Biofilm**—A group of microbes that grow together while adhering to each other and to a surface. Biofilms typically contain complex, diverse communities embedded in an extracellular, gelatinous matrix of polysaccharides, proteins, and DNA.

**Experimental microbial evolution**—A recently developed field of biology in which experiments with fast-growing and evolving populations of microorganisms are used to investigate evolutionary questions that cannot be addressed with slow-growing, larger organisms.

**Flexible genome**—The set of genes within a microbe's genome that are not ubiquitous in a species, but instead vary from strain to strain within that species. Typically, the flexible genome is larger than the core genome. Also called the dispensable, accessory, or adaptive genome.

**Gram-negative**—A diverse group of bacteria that have two membranes that regulate the entry of substances into and out of the cell, between which is a rigid cell wall that maintains the cell's shape and structural integrity. The name comes from the failure of these bacteria to retain crystal violet dye during the Gram-stain procedure.

**Hemolytic anemia**—Anemia caused by abnormal breakdown of red blood cells. In cases of *E. coli* O157:H7 infection, hemolytic anemia is caused by red blood cells being fragmented by blood clots that form in the capillaries.

**Microbiome**—The total microbial community that lives on and within the body of a large, multi-cellular organism like a human. The gut microbiome is typically by far the largest component of an organism's total microbiome.

**Pan-genome**—The complete set of all genes found among all strains of a microbial species.

**Pathotype**—A group of pathogenic strains of *E. coli* that cause disease in the same part of the body and via the same mechanism.

**Restriction Enzyme**—A DNA-degrading enzyme that recognizes and cleaves DNA at or near a particular sequence referred to as a ‘restriction site’. Bacteria produce restriction enzymes to defend against viruses by degrading their DNA upon its insertion into the cell. Also called a ‘restriction endonuclease’.

**Shiga-like Toxin**—A protein toxin produced by enterohemorrhagic *E. coli* that binds to particular receptors on the surfaces of epithelial cells in small blood vessels, mainly in the kidney, intestines, and lungs. Once in a cell, it inhibits protein synthesis and causes the cell to die (Griffin and Tauxe, 1991).

**Thrombocytopenia**—A lack of platelets in the blood, which reduces the ability of blood to clot. In *E. coli* O157:H7 infections, it is caused by large numbers of platelets being used up in small blood clots that form in the capillaries.

**Virome**—The sum total of all viruses that exist within or on an organism, including those within the microbiome, and those integrated into the organism's genome.

**DOI:** http://dx.doi.org/10.7554/eLife.05826.002

10.7554/eLife.05826.003

Box 2. **The contributions of *E.coli* to biology, medicine and industry**

Research using *E. coli* has led to many advances in a variety of fields. The following is a sample of these fields, and the contributions this work has made. Citations are non-exhaustive and to key literature only.

**Molecular Biology, Physiology, and Genetics**: Elucidation of the genetic code (Crick et al., 1961), DNA replication (Lehman et al., 1958), transcription (Stevens, 1960), life cycle of lytic and lysogenic bacterial viruses (Ellis and Delbrück, 1939; Lwoff, 1953), gene regulation (Jacob et al., 1960; Jacob and Monod, 1961; Englesberg et al., 1965), discovery of restriction enzymes (Linn and Arber, 1968; Meselson and Yuan, 1968), characterization and study of persister variants (Hu and Coates, 2005; Hansen et al., 2008; Lewis, 2010; Amato et al., 2013; Amato and Brynildsen, 2014) and swarming motility behavior (Harshey and Matsuyama, 1994; Harshey, 2003; Inoue et al., 2007; Partridge and Harshe, 2013a), and elucidation of the structure and function of ATP synthase (Capaldi et al., 2000).

**Pharmaceuticals**: In vivo synthesis of recombinant therapeutic proteins, including insulin (to treat diabetes), interleukin-2 (metastatic melanoma), human interferon-β (multiple sclerosis), erythropoietin (anemia), Human growth hormone (pituitary disorders, short stature, muscle wasting), human blood clotting factors (hemophilia), pegloticase (gout), taxol (cancer) and certolizumab (Crohn's disease) (reviewed in Kamionka, 2011; Huang et al., 2012).

**Evolution**: Demonstration of the random nature of mutations (Luria and Delbrück, 1943; Lederberg and Lederberg, 1952). Principal model organism in experimental evolution (reviewed in Kawecki et al., 2013), used to examine many issues, including the relationship between genomic evolution and adaptation (Barrick et al., 2009), evolutionary repeatability and the role of historical contingency in evolution (Travisano et al., 1995; Cooper et al., 2003; Blount et al., 2008; Meyer et al., 2012), the origin of novel traits (Blount et al., 2012), long-term fitness trajectories (Wiser et al., 2013), effect of sexual recombination on adaptation (Cooper, 2007), and predatory–prey interactions (Chao and Levin, 1977; Lenski, 1988; Meyer et al., 2010, 2012).

**Genetic Engineering and Biotechnology**: Development of genetic engineering techniques and technologies, including molecular cloning and recombinant DNA (Cohen et al., 1973), allele replacement (Link et al., 1997; Herring et al., 2003). Used to produce biofuels (Liu and Khosla, 2010; Janßen and Steinbüchel, 2014), and industrial chemicals such as phenol (Kim et al., 2014), ethanol (Hildebrand et al., 2013), mannitol (Kaup et al., 2004), and a variety of others (Chen et al., 2013).

**DOI:** http://dx.doi.org/10.7554/eLife.05826.003

**Figure 1.**
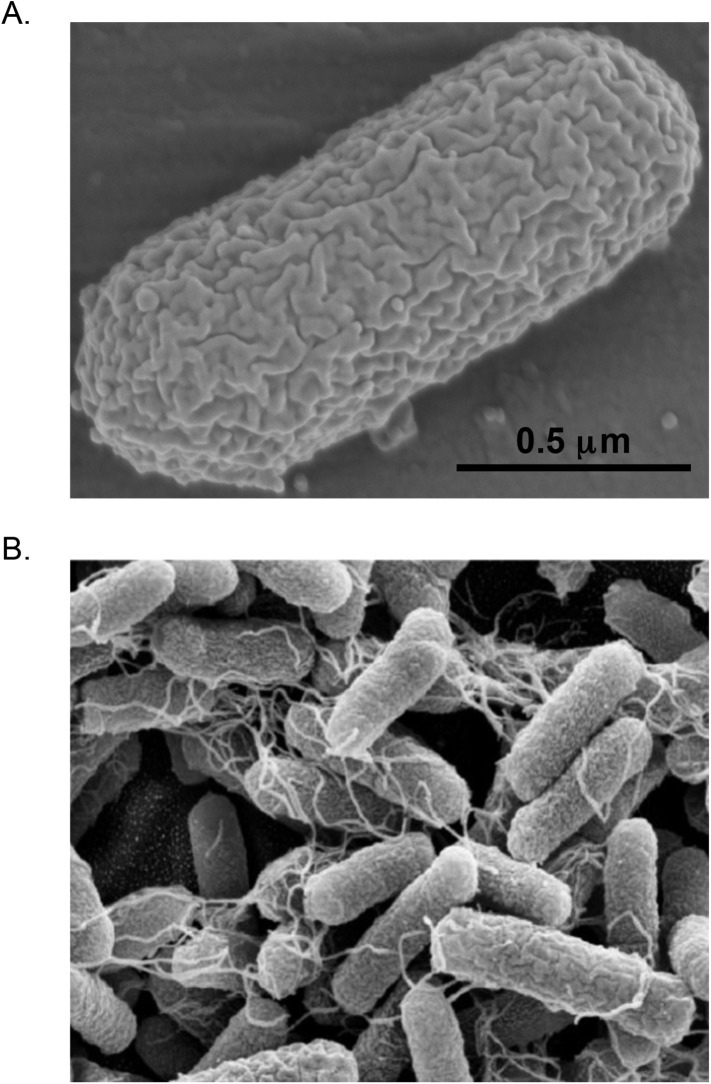
Scanning Electron Micrographs of *E. coli*. (**A**) *E. coli* B strain REL606, a laboratory strain with a typical sausage-shaped morphology. (Photo credit: Brian Wade). (**B**) *E. coli* O119:HND strain A111, an enteropathogenic strain that produces hair-like pili. (Photo credit: Nascimento et al., 2014). **DOI:** http://dx.doi.org/10.7554/eLife.05826.004

For all of its importance, *E. coli* is quite nondescript. It is a fairly typical Gram-negative bacillus (see ‘Glossary’), measuring only about 1 μm long by 0.35 μm wide, although this can vary considerably depending on the strain and its conditions. Even at high magnification it looks like nothing more than a tiny sausage (Figure 1A). It may have whip-like flagella that it uses to move about its environment, or hair-like pili that allow it to attach to surfaces or to other cells (Figure 1B). Physiologically, it is a facultative aerobe, meaning that it can grow happily with or without oxygen, but it cannot grow at extremes of temperature or pH nor can it degrade dangerous pollutants, photosynthesize, or do a variety of other things that interest microbiologists. Phylogenetically, it is a member of the Enterobacteriaceae, and is closely related to such pathogens as *Salmonella*, *Klebsiella*, *Serratia*, and the infamous *Yersinia pestis*, which causes plague (Brenner and Farmer, 2007).

## The helpful lodger: *E. coli*'s relationship(s) with its hosts

In nature, *E. coli* is principally a constituent of the mammalian gut microbiome (see ‘Glossary’), but it is also found, albeit less commonly, in the gut microbiomes of birds, reptiles and fish, as well as in soil, water, plants, and food (Hartl and Dykhuizen, 1984; Leimbach et al., 2013). Its mammalian abode is why *E. coli* can metabolize lactose, the control of which was the subject of seminal studies of gene regulation (Jacob et al., 1960; Jacob and Monod, 1961). *E. coli* is typically the most common aerobe in the lower intestine of mammals. However, the gut is primarily an anoxic environment, and the extremely large, highly diverse (500+ taxa) gut microbial community is dominated by obligate anaerobes, such as members of Bacteroides and Firmicutes, which alone comprise ∼90% or more of the total gut microbial population (Bäckhed et al., 2005; Eckburg et al., 2005; Claesson et al., 2009; Tenaillon et al., 2010). By contrast, *E. coli* typically constitutes only 0.1–5% of the community, which partly reflects the fact that its niche is to be found in the relatively thin layer of mucus that lines the gut. In the mucus layer, *E. coli* grows in a complex, multi-species biofilm (see ‘Glossary’) in which it competes for an array of nutrients—one of the origins of its broad diet (Chang et al., 2004; Beloin et al., 2008). This rich food supply enables *E. coli* to maintain population densities of 10^6^–10^9^ cells per gram of fecal matter despite unavoidable and regular bulk losses (Savageau, 1983; Chang et al., 2004). In the human gut, the *E. coli* population typically includes a set of long-term residential strains, and also short-term transients that vary with diet, health, and with exposure to antibiotics (Sears et al., 1950; Savageau, 1983). The human gut *E.coli* population also varies due to its constant, largely unknown interactions with the broader microbiome, and with the host and the host's vast virome (see ‘Glossary’), against which it defends itself with restriction enzymes, which have become a key tool in molecular biology (see ‘Glossary’) (Kasarjian et al., 2003; Roberts, 2005; Minot et al., 2011; He et al., 2013; Shoaie et al., 2013; Virgin, 2014).

A host organism carefully regulates its microbiome in poorly understood ways that have an impact on its health (Schluter and Foster, 2012). For instance, the human gut secretes immunoglobulin A, which appears to facilitate the formation of *E. coli* biofilms on the intestinal mucosa, suggesting that their presence is welcome (Bollinger et al., 2003). While long considered to have a commensal relationship with its host, in which *E. coli* secures food and a nice warm home while contributing little in return, it is increasingly clear that the host-*E. coli* relationship is really a mutualism. Indeed, *E. coli* benefits its host in a number of ways. It produces vitamin K and vitamin B12, both of which are required by mammalian hosts (Bentley and Meganathan, 1982; Lawrence and Roth, 1996). *E. coli* also maintains a friendly environment for its anaerobic neighbors by consuming oxygen that enters the gut. Perhaps most importantly, *E. coli* competitively excludes pathogens from its niche in the gut, rather like how friendly barbarian tribes, settled by the Roman Empire on its frontiers, helped to keep out the more dangerous tribes (Chang et al., 2004).

*E. coli*'s relationship with a host literally begins at birth. Newborns are typically inoculated with maternal *E. coli* through exposure to her fecal matter during birth and from subsequent handling (Nowrouzian et al., 2003; Leimbach et al., 2013). Although perhaps disconcerting to ponder, this inoculation seems to be quite important. Indeed, *E. coli* becomes more abundant in the mother's microbiome during pregnancy, increasing the chances of her newborn's inoculation (Koren et al., 2012). The colonizing strains typically have secretion systems and pili that allow them to attach to and interact with the infant's gut epithelium (Feeney et al., 1980; de Muinck et al., 2013). This newly established and rapidly growing *E. coli* population then changes the structure and function of the epithelial cells in ways that appear crucial for healthy microbiome development (Tomas et al., 2015). It is therefore concerning that early human infant colonization by *E. coli* has been declining in the US and in other Western nations as rates of caesarean delivery have increased and hospital hygiene has continued to improve (Grönlund et al., 1999; Nowrouzian et al., 2003; Adlerberth, 2006). Indeed, this decrease has been accompanied by broader microbiome changes, including increased infant gut colonization by *Staphylococcus aureus*, which is linked to an increased risk of developing a variety of disorders, including asthma, obesity, and diabetes (Lindberg et al., 2000, 2004; Neu and Rushing, 2011; Sannasiddappa et al., 2011; Rudi et al., 2012; Azad et al., 2013; Moeller et al., 2014). The long-term consequences of disrupting humanity's long association with *E. coli* are under intensive investigation.

## Life on the outside: *E. coli* in the external environment

An inevitable consequence of being a gut microbe is to be regularly excreted into the external world. The mucus lining of the gut is constantly sloughed off and excreted in fecal matter, so cells of a resident *E. coli* population are shed almost as soon as that population is established. *E. coli*'s long-term life cycle is hence biphasic, and despite being exquisitely adapted to the good life inside of a host, *E.coli* must also be adapted to successfully acclimate to a harsher life outside the host (Savageau, 1983). This is a remarkable feat. Whereas life on the inside is easy and stable, every aspect of the external environment, be it nutrition, temperature, oxygen, moisture, pH, and/or the surrounding microbial community, can fluctuate wildly (Savageau, 1983; Winfield and Groisman, 2003; van Elsas et al., 2011). It is likely that the hardiness, metabolic flexibility, and substrate breadth that have made *E. coli* such a valuable model organism evolved in part to permit it to survive this hostility and variability long enough to make it back to a host (van Elsas et al., 2011).

Another interesting trait that is almost certainly relevant to *E. coli*'s survival of its environmental phase is the production of persister variants. First observed in *Staphylococcus* during experiments with penicillin, persisters are rare, highly antibiotic-tolerant phenotypic variants that arise at random in bacterial populations (Hobby et al., 1942; Bigger, 1944; Balaban et al., 2004; Lewis, 2010; Zhang, 2014). Persisters are not an adaptation specifically to antibiotics; their hallmark antibiotic tolerance is attributable to their metabolic inactivity, which is triggered by several redundant pathways, including those that govern stress response (Shah et al., 2006; Lewis, 2010; Amato et al., 2013; Amato and Brynildsen, 2014). Persistence therefore appears to be a general adaptation that permits small numbers of dormant cells to survive a variety of environmental fluctuations. *E. coli* has been, as with so many other phenomena, a model in which to study bacterial persistence, and it seems likely that this capacity to enter a dormant state plays a significant role in surviving the considerable fluctuations it encounters in its external environment.

The external world was long thought to be so harsh as to preclude *E. coli*'s growth outside of its host. While a tiny minority might eventually reach a new host, most cells were expected to eventually die. This is the basal assumption behind using the presence of *E. coli* as an indicator of fecal contamination. However, recent studies have shown that *E. coli* can, in fact, establish itself as a member of microbial soil, water, and plant-associated communities (Lopez-Torres et al., 1987; Ishii and Sadowsky, 2008; Texier et al., 2008; Brennan et al., 2010; Berthe et al., 2013; Dublan et al., 2014). Moreover, genomic and phylogenetic analyses of collections of *E. coli* strains have identified divergent lineages that appear to be adapted to a primarily non-host lifestyle (Walk et al., 2009). What adaptations are required for *E. coli* to make such radical ecological shifts, what niches it fills in its new communities, how stable its presence in those communities might be, and what impact its adaptation to these new niches might have on its capacity to return to a host remain outstanding questions that must be addressed (see Box 3).

10.7554/eLife.05826.005

Box 3. **Outstanding questions about the natural history of *E. coli***

- How do *E. coli* populations become established in soil and water communities outside of their host? What niches do they fill? How does adaptation to these new conditions affect their capacity to recolonize a host organism?
- How often do environmental strains find their way back to a host?
- What is the function of swarming motility in nature?
- What proportion of any given *E. coli* strain's complement of genes is adaptive to life in a host vs life in the external environment?
- How many different ecotypes of *E. coli* that occupy distinct niches are there?
- To what degree is *E. coli*'s genomic evolution in the wild driven by horizontal gene transfer vs mutation? How much horizontal gene transfer into *E. coli* comes from other organisms?
- How do the *E. coli* pan- and flexible genomes evolve, and what overlap is there with those of other organisms?
- On average, how many generations do wild *E. coli* strains undergo in a year?
- What is the average pace of evolution for wild *E. coli*? How does it vary between different strains occupying different hosts and environments?
- How tightly have *E. coli* and its hosts co-evolved?
- What impact does change in human lifestyles have on the relationship between humans and *E. coli*, and what are the health consequences?

**DOI:** http://dx.doi.org/10.7554/eLife.05826.005

It is possible that these environmental *E. coli* populations will help resolve the interesting problem of swarming motility. It has long been observed that groups of *E. coli* cells on water-restricted surfaces will congregate and engage in social, coordinated movement over the surface, a behavior also seen in other bacteria (Harshey and Matsuyama, 1994; Harshey, 2003; Partridge and Harshey, 2013a,b). However, swarming has principally been observed and studied on agar plates in the lab (Partridge and Harshey, 2013a). What function it might serve for *E. coli* in nature has been unclear. The gut generally lacks the sorts of surfaces on which swarming works. Given that ∼216 *E. coli* genes are specifically involved in swarming motility, it is a costly trait that would not be maintained if it did not confer some selective benefit (Inoue et al., 2007). It is as yet unclear if swarming plays any role in the initial colonization of the gut, but it is associated with pathogenesis, likely due to its improved colonization of tissue surfaces that facilitate opportunistic infection (see the next section) (Zhang et al., 2010; Partridge and Harshey, 2013a). Given that surfaces, and interfaces between surfaces, are regularly encountered by environmental bacteria, swarming may well prove to be an adaptation that enables the exploration and colonization of viable habitats by *E. coli* upon their excretion into the external environment.

## Pathogenic *E. coli*: A friendly microbe's dark side

*E. coli*'s presence in the environment is a cause for concern because its relationship with humans is not entirely benign. Indeed, *E. coli* is a major cause of diarrheal diseases, peritonitis, colitis, bacteremia, infant mortality, and urinary tract infections that world-wide cost billions of dollars to treat and kill roughly 2 million humans each year (Russo and Johnson, 2003; Kaper et al., 2004). Some strains may even cause cancer (Arthur et al., 2012). Some opportunistic *E.coli* infections are caused by normally harmless or beneficial strains when introduced to sick hosts or to parts of a host's body outside of the gut (Kaper et al., 2004). However, there are also pathogenic strains that produce virulence factors and can cause illness in even the healthiest host. These strains are classified by where and how they cause disease into groups called pathotypes (see ‘Glossary’), which include enteroaggregative, enterohemorrhagic, enteropathogenic, enterotoxigenic, uropathogenic, meningitis-associated, and septicemic-associated *E. coli* (Kaper et al., 2004; Leimbach et al., 2013) (see ‘Glossary’).

The most notorious of these is *E. coli* O157:H7, an enterohemorrhagic strain that produces a shiga**-**like toxin (Griffin and Tauxe, 1991; Robinson et al., 2006). This toxin (see ‘Glossary’) attacks small blood vessels, killing intestinal cells, and causing bloody diarrhea and severe abdominal pain, as well as hemolytic uremic syndrome (HUS), a potentially deadly condition that can involve widespread clots in capillaries and hemolytic anemia, thrombocytopenia, and renal failure (see ‘Glossary’; Griffin et al., 1988; Kaper et al., 2004). Treatment can be difficult because antibiotics increase the risk of HUS. As a result, treatment is generally limited to the provision of fluids, adequate nutrition, medication for pain and fever, and blood transfusions when necessary (Bitzan, 2009; Smith et al., 2012).

O157:H7 is particularly dangerous because it can easily contaminate human food supplies. It resides asymptomatically in cattle and in other livestock, and can be transferred to humans via the fecal contamination of meat during its butchering and packaging (Ferens and Hovde, 2011). It can also contaminate vegetables via fertilizers and water, and through contact with live-stock-associated birds (Callaway et al., 2009, 2014). Through these points of entry into the human food chain, O157 has caused numerous outbreaks of illness (Frenzen et al., 2005; Rangel et al., 2005). In the US alone, such outbreaks have annually affected ∼63,000 individuals, killing 20, and costing around $405 million in healthcare and in lost productivity (Mead et al., 1999; Scallan et al., 2011). When *E. coli* gets bad press, O157:H7 is almost always the culprit, and rightfully so.

## Diversity and plasticity of the *E. coli* genome

The many pathogenic strains of *E. coli* testify to the diversity of this single microbial ‘species’, the full extent of which was only revealed by the advent of whole-genome sequencing. For example, genome sequences have firmly placed all *Shigella* strains within the broader *E. coli* clade (Pupo et al., 2000; Lukjancenko et al., 2010; Kaas et al., 2012).

More importantly, sequencing has uncovered the remarkable plasticity and dynamism of the *E. coli* genome that contribute to its genetic and phenotypic diversity. In 2002, Welch et al. (2002) reported that three strains of *E. coli*, the popular lab strain K-12, O157:H7, and the uropathogenic strain CFT073, share only 39.2% of their genes. Subsequent sequencing of more strains has reduced this core genome to less than 20% of the more than 16,000 genes in the *E. coli* pan-genome (see ‘Glossary’; Lukjancenko et al., 2010; Kaas et al., 2012).

The remaining genes constitute the flexible genome (see ‘Glossary’), a vast pool of ‘plug and play’ genetic variation that can be acquired via horizontal gene transfer (Lukjancenko et al., 2010; Mira et al., 2010; Leimbach et al., 2013). This variation includes prophages, transposable elements, and accessory genes (see ‘Glossary’). These genes encode functions that can: improve fitness in particular niches; increase metabolic flexibility; and affect pathogenicity (Schmidt and Hensel, 2004; Touchon et al., 2009). Flexible genomic elements are often large and integrate into the genome at select insertion hotspots (Touchon et al., 2009). This capacity to mix and match accessory genetic elements means that new *E.coli* strains with novel combinations of traits can arise very quickly. Another consequence is that the size of the *E. coli* genome can vary greatly between strains. While standard lab strains have genomes of ∼4.5 million base pairs and 4000 genes, pathogenic strains can have genomes of over 5.9 million base pairs and 5500 genes (Blattner et al., 1997; Lukjancenko et al., 2010; de Muinck et al., 2013).

This extensive genetic plasticity poses major questions for understanding how *E. coli* evolves in the wild over the long-term. For instance, how does the rate of genomic evolution in a given *E. coli* lineage by mutation compare to that by horizontal gene transfer (Guttman and Dykhuizen, 1994; Dolbrindt et al., 2010; Paul et al., 2013)? Moreover, how much of the *E. coli* flexible genome ultimately derives from other organisms?

## Conclusion

*E. coli* has been tremendously valuable as a de-contextualized, abstracted model organism, but it could be of even greater value were we to gain a better understanding of its ecology and natural history. As I have discussed, it is a highly diverse and broadly distributed species that occupies an expansive, generalized niche in which it experiences a vast range of environmental and ecological conditions. This breadth presents two unique and synergistic opportunities. First, the tools and techniques developed for, and the knowledge derived from, the study of *E. coli*'s lab strains can be applied to studying its wild relatives in far greater detail than is possible for any other microbe. This capacity promises to yield new and profound insights into the biology of other microbes that experience similar conditions, as well as the discovery and identification of new microbiological phenomena. Second, any increase in our knowledge of *E. coli*'s natural history expands the range of biological phenomena for which its strains can be used as models to study. In other words, the study of *E. coli*'s natural history can reveal more than is possible with most organisms, and an increased understanding of its natural history in turn expands its potential as a model organism.

An example of this dynamic can be seen in studies of *E. coli* biofilms, which have revealed much about how harmless and pathogenic strains colonize the gut and persist in the environment (Wang et al., 2006; Beloin et al., 2008; Nesse et al., 2014; Vogeleer et al., 2014). These studies have, in turn, led to the development of *E. coli* as a model for studying the formation, genetics, physiology, and consequences of biofilms, generating important findings on this most common of microbial lifestyles (Beloin et al., 2008). Experimental evolutionary studies of *E. coli* biofilms have also led to findings of far-reaching consequence, such as how biofilms impact microbial evolution and how they facilitate the evolution of antibiotic resistance even when antibiotics are not present (Ponciano et al., 2009; Tyerman et al., 2013).

There are many unanswered questions about *E. coli*'s natural history (see Box 3). How does *E. coli* adapt to non-host environments? What role does it play in non-host communities? How does it adapt to life in the soil? Just how fluid is the *E. coli* genome? How do environmental and ecological conditions affect this fluidity? How does the pan-genome evolve? How much interaction is there between *E. coli*'s pan-genome and that of other microbes? How tightly adapted are hosts to their *E. coli* populations? How is *E. coli* adapting to changes in the human diet and lifestyle? Each unanswered question presents opportunities for novel research into unexplored corners of *E. coli*'s natural history, and the subsequent expansion of its potential as a model. Improved appreciation and interest in *E. coli*'s natural history can only uncover more questions, and increase its potential even more. If kept in mind by researchers, this dynamic will guarantee that the most important model organism of the 20^th^ century will continue to be one of the most important model organisms of the 21^st^ century and beyond.
